# Decision-making based on 3D printed models in laparoscopic liver resections with intraoperative ultrasound: a prospective observational study

**DOI:** 10.1007/s00330-019-06511-2

**Published:** 2019-11-26

**Authors:** Jan Witowski, Andrzej Budzyński, Anna Grochowska, David H. Ballard, Piotr Major, Mateusz Rubinkiewicz, Adriana Złahoda-Huzior, Tadeusz J. Popiela, Mateusz Wierdak, Michał Pędziwiatr

**Affiliations:** 1 2nd Department of General Surgery, Jagiellonian University Medical College, Kopernika 21, 31-501 Krakow, Poland; 2 Center for Research, Training and Innovation in Surgery (CERTAIN Surgery), Krakow, Poland; 3 Department of Radiology, Jagiellonian University Medical College, Kopernika 19, 31-501 Krakow, Poland; 4 Mallinckrodt Institute of Radiology, Washington University School of Medicine, St. Louis, MO 63110 USA; 5 Department of Measurement and Electronics, AGH University of Science and Technology, Al. Mickiewicza 30, 30-065 Krakow, Poland

**Keywords:** 3D printing, Liver cancer, Hepatectomy, Decision-making, Ultrasonography

## Abstract

**Objectives:**

The aim of this study was to evaluate impact of 3D printed models on decision-making in context of laparoscopic liver resections (LLR) performed with intraoperative ultrasound (IOUS) guidance.

**Methods:**

Nineteen patients with liver malignances (74% were colorectal cancer metastases) were prospectively qualified for LLR or radiofrequency ablation in a single center from April 2017 to December 2018. Models were 3DP in all cases based on CT and facilitated optical visualization of tumors’ relationships with portal and hepatic veins. Planned surgical extent and its changes were tracked after CT analysis and 3D model inspection, as well as intraoperatively using IOUS.

**Results:**

Nineteen patients were included in the analysis. Information from either 3DP or IOUS led to changes in the planned surgical approach in 13/19 (68%) patients. In 5/19 (26%) patients, the 3DP model altered the plan of the surgery preoperatively. In 4/19 (21%) patients, 3DP independently changed the approach. In one patient, IOUS modified the plan post-3DP. In 8/19 (42%) patients, 3DP model did not change the approach, whereas IOUS did. In total, IOUS altered surgical plans in 9 (47%) cases. Most of those changes (6/9; 67%) were caused by detection of additional lesions not visible on CT and 3DP.

**Conclusions:**

3DP can be helpful in planning complex and major LLRs and led to changes in surgical approach in 26.3% (5/19 patients) in our series. 3DP may serve as a useful adjunct to IOUS.

**Key Points:**

*• 3D printing can help in decision-making before major and complex resections in patients with liver cancer.*

*• In 5/19 patients, 3D printed model altered surgical plan preoperatively.*

*• Most surgical plan changes based on intraoperative ultrasonography were caused by detection of additional lesions not visible on CT and 3D model.*

## Introduction

Laparoscopic liver resection (LLR) has been widely adopted as an acceptable and often preferable approach for resections in patients with hepatic malignancies, including colorectal intrahepatic metastases, over the last 20 years. Recent meta-analyses and studies, including OSLO-COMET randomized controlled trial, proved LLRs to be non-inferior and in many cases superior to open surgery, especially in terms of reduced blood loss, lower or similar morbidity, and comparable mortality and survival [1–5]. A minimally invasive approach comes, however, with some limitations. Magnified view, which is considered a great advantage of laparoscopy, also disrupts the natural perception of anatomy and restricts the overview. Additionally, lack of tactile sensation is often described as a reason for surgeons’ disorientation during the procedure [6]. This is especially true in more difficult resections, including sectionectomies of lesions in postero-superior locations that require extensive liver mobilization and work with not fully satisfactory field of view [7]. To overcome these obstacles, intraoperative ultrasound (IOUS) has been widely used. IOUS is now strongly recommended by the 2018 Southampton Consensus Guidelines to be available in every LLR case, as it can potentially help in planning the resection line and precise tumor location [8]. Having said that, it would be preferable for IOUS to only confirm or slightly alter preoperative findings, with an accurate surgical plan having been established prior to the procedure. For this reason, advancements in presurgical imaging are explored. They, however, have often been burdened by high costs of proprietary software or poor rendering quality. Moreover, images presented by specialized software are still assessed on flat 2D screen. The rise of modern 3D visualization techniques, including 3D printing (3DP) and augmented reality, tries to present the models in more realistic ways, allowing the surgeon haptic feedback and real-time visualization. 3DP has been employed in recent years to support clinicians from various fields and was found to be beneficial in terms of perioperative outcomes [9–11]. There is a major gap in literature regarding how 3DP models affect decision-making and, most importantly, clinical outcomes. Although a few recent publications attempted to quantitatively assess 3DP impact, most research provided conclusions based on questionnaires and subjective results [12]. This is also the case with the field of liver resections as no prospective studies have been published so far, especially involving IOUS use.

The aim of this study was to evaluate impact of 3D printed models on decision-making in context of LLRs performed with IOUS guidance.

## Materials and methods

This study was approved by the local research Ethical Committee following the guidelines of the Declaration of Helsinki of 1975 with its later revisions. All subjects gave their informed consent for inclusion before they participated in the study. This research is a part of a trial registered in ClinicalTrials.gov database under identification number NCT03744624. The clinical trial is expected to enroll 85 patients by December 2022; thus, this study can be considered a preliminary study that allows continuing with the protocol. We followed a cost-effective method of developing full-sized 3DP liver models introduced by Witowski et al [13]. Accuracy of this approach has been previously confirmed [14]. Fifteen out of 19 patients have been previously reported in the latter study. This prior article dealt with investigating precision of models, whereas in this manuscript we report on clinical impact.

Consecutive patients with liver tumors were qualified to undergo LLR or radiofrequency ablation (RFA) with the use of IOUS from April 2017 to December 2018 in a tertiary referral center and prospectively included to study. Study participants’ characteristics are presented in Table 1. Initially, surgery was planned after CT assessment made by a multidisciplinary team including surgeons and radiologists. Subsequently, a personalized model was 3D printed and delivered to surgical team at least 5 days prior to the procedure. After reviewing 3DP model and discussing it within the team, primary decision about surgery extent was reviewed and any changes were noted. Finally, the ultimate decision taken intraoperatively with the use of IOUS was recorded. IOUS was considered a reference standard.

**Table 1:** Study participants’ characteristics and surgical details

Parameters	(*N* = 19)
Male/female	13/6
Age, median (range), y	64.5 (35–81)
BMI, mean ± SD	26.2 ± 3.7
Child-Pugh A/B	17/2
mCRC/HCC/SFT	14/4/1

*mCRC* colorectal liver metastases, *HCC* hepatocellular carcinoma, *SFT* solitary fibrous tumor

Nineteen participants were included into analysis and, based on CT evaluation, 16 were qualified for LLR, 2 participants for RFA, and in one case a laparoscopic associating liver partition and portal vein ligation for staged hepatectomy (ALLPS) procedure was planned. Median operative time was 210 min (range 30–540 min) and median blood loss 450 mL (range 0–2600). There have been two conversions to open surgery.

### Imaging

All images were acquired according to the clinical protocol on a 64-slice GE Optima CT660 system (GE Medical Systems). CT protocol included pre- and post-contract imaging in arterial, hepatic/portal, and delayed phases. Hepatic phase, which was used for segmentation, was acquired 30 s after arterial phase (approximately 75 s total delay). Parameters of acquired axial images were slice thickness of 1.25 mm, 120 kVp, total collimation width 40 mm, table feed per rotation 39.375 mm, and 512 × 512 matrix.

### 3D printing

Detailed technical note of model development was described previously [13]. Compared to the original methodology, we have been using 3D Slicer software (open source; slicer.org) for segmentation instead of Horos (open source; horosproject.org) [15]. Segmentation was performed by J.W. and reviewed by A.G. (radiologist with 15-year experience in abdominopelvic imaging). Segmentation used semi-automatic algorithms including region growing and thresholding to create initial regions of interests. They were then corrected with manual tools such as paint brush/eraser or prebuilt level tracing for pixel-level accuracy. It is important to mention that this part of methodology is crucial to ensure printed model accuracy.

**Fig. 1:**
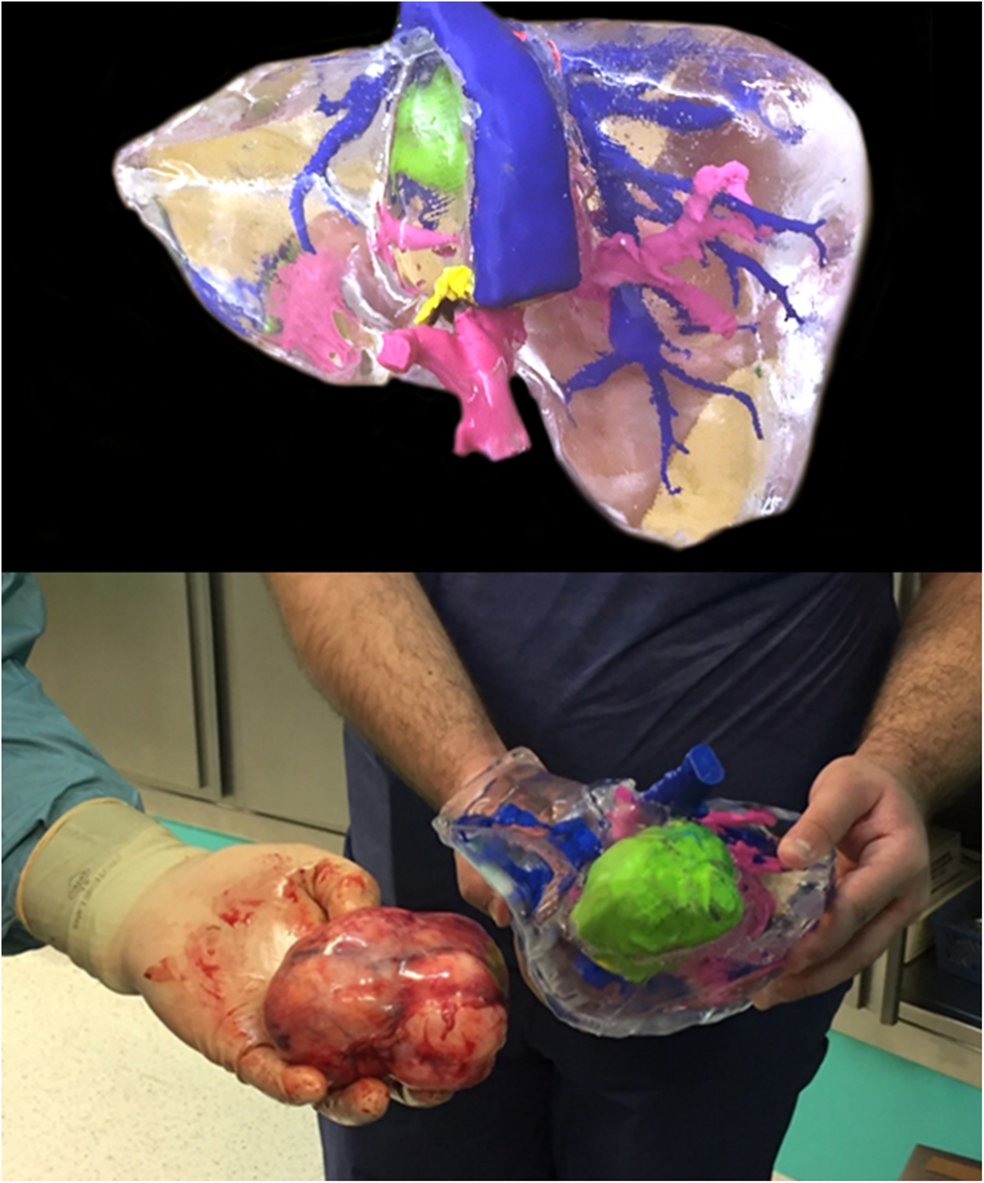
Examples of 3D printed models used for preoperative planning. Life-sized models were developed with by standard 3D printing and silicone casting. Structures were marked as follows: green, lesions; magenta, portal veins; blue, hepatic veins with inferior vena cava

Segmented parts of hepatic and portal veins, liver parenchyma, and tumors were 3D printed using Ultimaker 2+ printer (Ultimaker) with polylactic acid filaments. Developed parts were assembled to create a mold, which was smoothed and coated with resin and subsequently casted with transparent silicone. In comparison to primary development technique, we also reduced silicone curing time from 72 to 48 h and replaced bonding printed parts using cyanoacrylate adhesive with plastic friction welding due to problems with silicone leaking. In most cases, process of preparing 3DP models requires at least five working days (Fig. 1).

### Endpoints

Primary endpoints of this study were changes in the extent of surgical resection, moments when decisions have been made and their significance. Changes were considered major if they involved more than one liver segment in accordance to Couinaud’s classification (considering segments 4a and 4b as two separate segments). Data were stratified by 3DP models changing surgical approach without any further change from IOUS, 3DP models changing approach altered later by IOUS, and circumstances where 3DP did not identify a change in approach whether IOUS did.

## Results

**Fig. 2:**
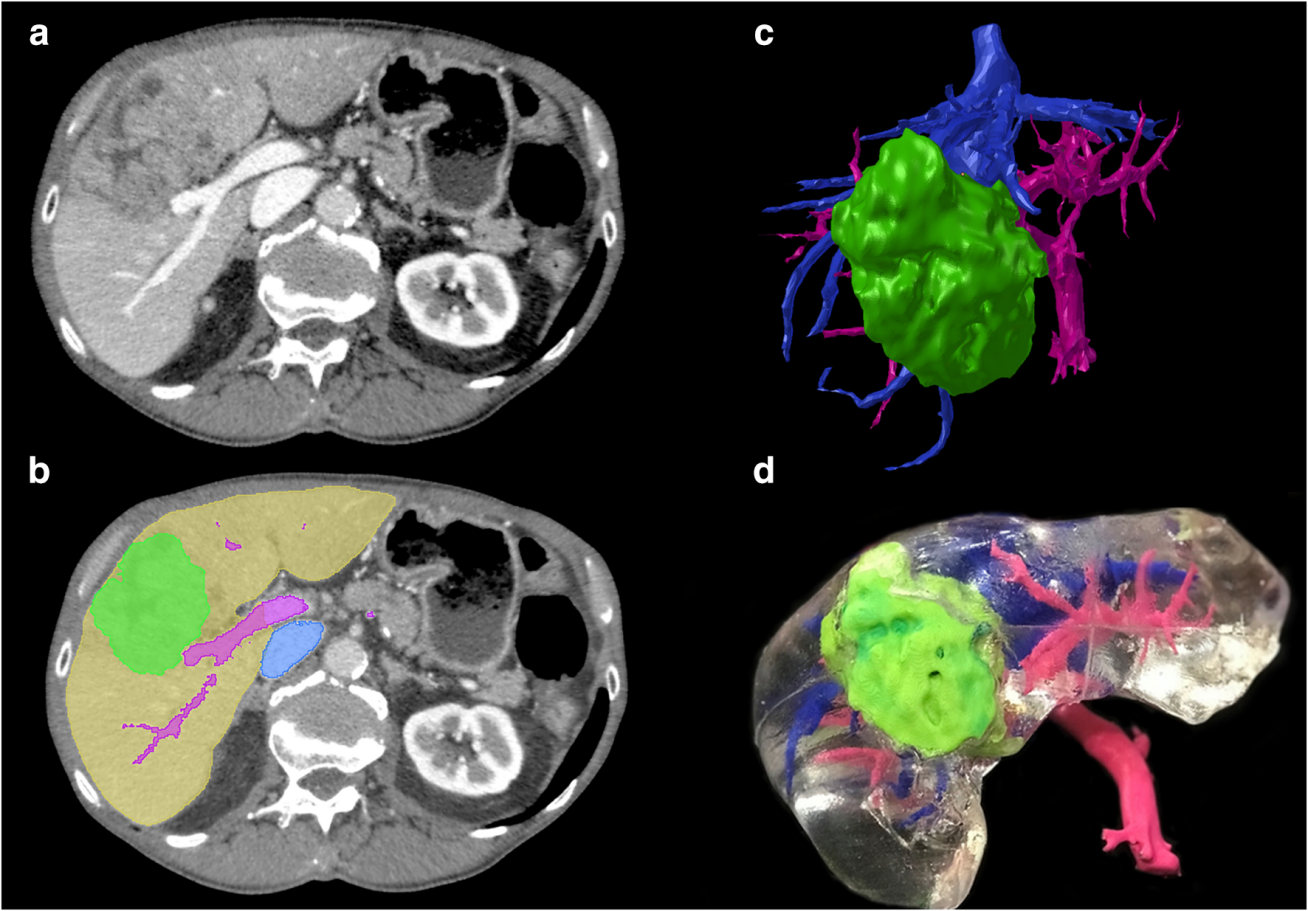
Development of 3D printed liver models. **a** Raw CT axial image; **b** CT image with segmentation masks overlay (green, tumor; yellow, parenchyma; pink, portal vein; blue, inferior vena cava); **c** Surface rendering of segmented meshes; **d** 3D printed liver model

3D printed models were developed, delivered, and used by surgical team as planned in study design (Fig. 2). In 5/19 (26.3%) patients, surgical plan was changed after examination of 3D liver models and four of these changes (21% of all patients) were considered major (Table 2). In 4/19 (21%) patients, 3DP model changed the surgical plan which was not further altered by IOUS. In one (5.3%) patient, after 3DP changed the approach, it was modified again after IOUS. In 8/19 (42.1%) patients, 3DP model did not change the approach, whereas IOUS did.

**Table 2:** Extent of liver resections planned by surgical team after analysis of patient imaging and 3D printed model, compared to final decision. Patients in table are stratified into four groups, depending on reasons for the change of the surgical plan. Final surgical plan was decided intraoperatively with the assistance of laparoscopic ultrasound

**#**	Surgical plan based on CT	Surgical plan based on 3D printed model	Final surgical plan (after intraoperative ultrasound)
Changes made after 3DP			
1	Left hepatectomy	Extended left hepatectomy*	Extended left hepatectomy (conversion)
2	Left hepatectomy	Segment 1 anatomic resection*	Segment 1 anatomic resection
3	Right hepatectomy	Extended right hepatectomy*	Extended right hepatectomy
4	Extended right hepatectomy	Central hepatectomy*	Central hepatectomy
Changes made after 3DP and after IOUS			
5	Segment 6 anatomic resection	*Non-anatomic resection* (*segment 6 with a fragment of segment 7*)*	*Non-anatomic resection* (*segment 6 with fragments of segments 5 and 7*)*
Changes made after IOUS			
6	RFA of a single lesion	RFA of a single lesion	Non-anatomic resection*
7	RFA of two lesions	RFA of two lesions	RFA of three lesions*
8	ALLPS	ALLPS	*Disqualified from ALLPS* (*dissemination*)*
9	Non-anatomic resection	Non-anatomic resection	*Disqualified from resection* (*dissemination*)*
10	Segment 8 anatomic resection	Segment 8 anatomic resection	Segment 8 anatomic resection with removal of two minor superficial lesions on segments 5, 6*
11	Segments 6, 7 bisegmentectomy	Segments 6, 7 bisegmentectomy	Right hepatectomy*
12	Segments 2, 3 bisegmentectomy with non-anatomic segment 5 resection	Segments 2,3 bisegmentectomy with non-anatomic segment 5 resection	Two non-anatomic resections*
13	Right hepatectomy	Right hepatectomy	Right hepatectomy with segment 3 metastasectomy*
No changes were made			
14	Local resection with gallbladder	Local resection with gallbladder	Local resection with gallbladder
15	Non-anatomic resection	Non-anatomic resection	Non-anatomic resection
16	Non-anatomic resection	Non-anatomic resection	Non-anatomic resection
17	Two metastasectomies	Two metastasectomies	Two metastasectomies
18	Right hepatectomy	Right hepatectomy	Right hepatectomy
19	Right hepatectomy with segment 2 metastasectomy	Right hepatectomy with segment 2 metastasectomy	Right hepatectomy with segment 2 metastasectomy (conversion)

Asterisks (*) and text in italics denote change of surgical approach

*IOUS* intraoperative ultrasound, *mCRC* metastatic colorectal cancer, *HCC* hepatocellular carcinoma, *ALLPS* associating liver partition and portal vein ligation for staged hepatectomy, *RFA* radiofrequency ablation

In total, after IOUS guidance, decision about surgical extent was altered in 9/19 (47.4%) patients, including two situations where patients were disqualified from the surgery. Majority of these changes—6 out of 9 (66.7%)—involved a maximum of one anatomical segment. Six surgical plan changes based on IOUS included resection or RFA of additional lesions that were not visible on preoperative imaging (patients 5, 6, 7, 10, 11, 13).

## Discussion

In our study, we have shown that 3DP models used preoperatively can change the surgical plan, in some cases altering the surgery extent significantly. It seems that 3DP models are especially useful as a tool for general visual assessment of tumor location and “visual volumetry.” This approach to roughly estimating residual liver volume and predicting preserved liver function can be found appropriate in patients undergoing major or complex LLRs.

Out of around 20 research papers that have been published in the field of 3DP in liver surgery, none assess quantitatively how models affect decision-making and most of them lack description about clinical use [16, 17]. Igami et al displayed feasibility of using 3DP models in choosing liver partition line [18] and earlier in determining resection line before small hepatectomy of tumor invisible in IOUS [19]. Selecting and evaluating optimal resection line was also performed by Oshiro et al who confirmed benefits of this approach [20]. These publications, however, did not lead to measurable change in the operative approach. Our study builds up on that initial evidence, suggesting that 3DP can help not only in choice of resection line but in determining surgical extent. Also, we demonstrated clinical feasibility as all of the models were used prospectively in patient care.

Role and diagnostic accuracy of both CT and IOUS in LLRs have been widely explored in literature. IOUS greatest value lies in providing surgeons with real-time control during resection and higher than preoperative CT diagnostic accuracy in regards to detecting lesions, with its sensitivity estimating around 90% [21–23]. Ferrero et al found that the use of IOUS changed surgical strategy in 27.2% of all cases, out of which 83.6% changes were due to new lesions detected by IOUS [22]. New tumors explored with IOUS are usually smaller than 10 mm [22, 24, 25]. Detection of new lesions was also noticeable in our study, as it was a reason for most intraoperative decision changes. 3DP models will not detect new lesions and their diagnostic sensitivity can be as high as primary imaging. Still, our study showed that in 26.3% of patients, surgical plan was changed, even without new malignancies. This can be because of better insight into spatial relationships between lesions and liver vasculature. These relationships can possibly be understood based on CT analysis only, but it strictly depends on surgeon’s knowledge of radiological anatomy. 3DP models, however, do not require considerable experience to quickly, easily, and fully comprehend vascular structure in each case, which makes them potentially more approachable in operative planning. Additionally, it is worth mentioning that IOUS never reverted the decision about surgery extent to the initial one after CT analysis.

Reports also suggest IOUS being beneficial in terms of identifying boundaries of resection intraoperatively [26–28]. Frankly, to our best knowledge, these differences have not been quantitatively researched so far and our series is a start to this evaluation.

In our opinion, the feasibility of 3D liver models lays in the proper planning of the extent of liver resection. It is crucial in patients undergoing major liver resections in order to detect candidates who may suffer from post-hepatectomy liver failure. Isolating that cohort can allow clinicians to implement additional safety measures prior to the procedure and immediately after. Four out of five decision changes based on 3DP in our study involved more than one segment, suggesting this approach could potentially assist in detecting patients at risk.

Aforementioned changes were usually a result of change in cognitive localization of tumors in the liver. Although our study did not prove this fundamentally, recently published research by Wake et al shows that understanding renal tumor location based solely on CT is poor and significantly improved with 3DP models [29]. Igami et al 2014 research also suggested that with 3DP liver models surgeons were able to better comprehend spatial relationships, although with no quantitative results [19]. Our experience supports those results and we believe they could be reproduced with liver tumors. In this study, we focus on the use of computed tomography. However, methodology is fully applicable to MRI datasets. The feasibility of 3DP liver models based on MRI was previously reported by Ripley et al [30].

There are several limitations of our study. First of all, our study does not have a control group without the use of 3DP models, which makes it difficult to perform inferential statistics comparing how IOUS itself would change the extent of resection in patients without newly found lesions. Rather, the data presented in this series reflects an institutional practice change in using 3DP anatomic model prospectively in patient’s care. Also, there are a limited number of patients included in the study who underwent LLR for primarily (*n* = 15/19; 79%) colorectal intrahepatic metastasis, making it difficult to generalize the results to patients with other intrahepatic malignancies. Our study did not look at treatment short- and long-term outcomes. This should be a subject for larger, preferably multi-institutional trials that enroll a sufficient number of patients to show statistically significant results. It is also important to take action towards quality assurance and methodology standardization as results of 3DP are strongly dependent on segmentation. Future studies should also look at educational aspects of liver models, both for students and surgeons as well as for patients, as this has been suggested to be a beneficial area [31–33]. They could also potentially serve as quality control devices for tumor resections, discussing approach or feasibility of the surgery, among other possibilities.

3DP in liver surgery has more challenges ahead: it is still a relatively expensive method, although use of low-cost approaches, as suggested by Witowski et al or Watson, could help with access to this technology [13, 34]. Alternatively, other 3D visualization techniques including realistic 3D surface rendering or augmented/virtual reality should be explored and directly compared to 3DP models in future studies [31]. 3DP needs to be adopted to new surgical techniques, such as ALLPS (associating liver partition with portal vein ligation for staged hepatectomy), and synergized with new imaging methods in surgery, including intraoperative fluorescence, contrast-enhanced intraoperative ultrasound, or co-registration between intraoperative ultrasound and MRI/CT [35–38].

3DP liver models are feasible in planning LLRs and can help in establishing surgical plan in major and complex resections, occasionally changing surgical approach by more than one segment. This may be especially promising in situations where IOUS is not available. However, 3DP models will not increase sensitivity of lesion detection, which confirms the necessity of IOUS routine use.

## Abbreviations

3DP: 3D printing
ALLPS: Associating liver partition and portal vein ligation for staged hepatectomy
IOUS: Intraoperative ultrasound
LLR: Laparoscopic liver resection
